# The Inflammasome Contributes to Depletion of the Ovarian Reserve During Aging in Mice

**DOI:** 10.3389/fcell.2020.628473

**Published:** 2021-02-11

**Authors:** Carolina Lliberos, Seng H. Liew, Ashley Mansell, Karla J. Hutt

**Affiliations:** ^1^Department of Anatomy and Developmental Biology, Monash Biomedicine Discovery Institute, Monash University, Clayton, VIC, Australia; ^2^Centre for Innate Immunity and Infectious Diseases, Hudson Institute of Medical Research, Clayton, VIC, Australia

**Keywords:** inflammation, inflammasome, follicle, ovary, ovarian ageing, cytokine, NLRP3, ASC

## Abstract

Ovarian aging is a natural process characterized by follicular depletion and a reduction in oocyte quality, resulting in loss of ovarian function, cycle irregularity and eventually infertility and menopause. The factors that contribute to ovarian aging have not been fully characterized. Activation of the NLRP3 inflammasome has been implicated in age-associated inflammation and diminished function in several organs. In this study, we used *Asc*^−/−^ and *Nlrp3*^−/−^ mice to investigate the possibility that chronic low-grade systemic inflammation mediated by the inflammasome contributes to diminished ovarian reserves as females age. Pro-inflammatory cytokines, IL-6, IL-18, and TNF-α, were decreased in the serum of aging *Asc*^−/−^ mice compared to WT. Within the ovary of reproductively aged *Asc*^−/−^ mice, mRNA levels of major pro-inflammatory genes Tnfa, Il1a, and Il1b were decreased, and macrophage infiltration was reduced compared to age-matched WT controls. Notably, suppression of the inflammatory phenotype in *Asc*^−/−^ mice was associated with retention of follicular reserves during reproductive aging. Similarly, the expression of intra-ovarian pro-inflammatory cytokines was reduced, and follicle numbers were significantly elevated, in aging *Nlrp3*^−/−^ mice compared to WT controls. These data suggest that inflammasome-dependent inflammation contributes to the age-associated depletion of follicles and raises the possibility that ovarian aging could be delayed, and fertile window prolonged, by suppressing inflammatory processes in the ovary.

## Introduction

Female fertility declines dramatically with age, primarily due to the loss of oocyte number and quality (Findlay et al., 2018). A woman's lifetime supply of oocytes is stored in her ovaries in structures called primordial follicles. Most primordial follicles are dormant, but a few at a time become activated to begin folliculogenesis, which is characterized by growth of the oocyte, plus the proliferation, and differentiation of the granulosa cells that support oocyte development. Folliculogenesis results in the cyclic production of female reproductive hormones that have important roles in pregnancy and female health, and ultimately culminates in the ovulation of a mature oocyte. After birth, the number of primordial follicles steadily declines as consequence of follicle recruitment, follicle atresia, and the normal aging process. Eventually the supply of healthy follicles becomes so low that females become infertile and undergo menopause. In addition to natural ovarian aging, follicles can be depleted much earlier than expected, leading to premature ovarian insufficiency. Loss of ovarian function, as a consequence of either physiological or pathological processes, has serious and widespread health impacts for women, including infertility and increased risk of heart disease and osteoporotic fractures because of reduced hormone production (Broekmans et al., 2009). Despite this significance, the suite of molecular mechanisms underlying the normal age-specific decline in ovarian function, or the early loss of ovarian follicles, remain largely unknown.

Non-infectious chronic, low-grade inflammation, is a hallmark of the normal aging process, that underpins a range of age-related pathologies (Franceschi et al., 2000). It is proposed that damaged cells and macromolecules that accumulate with age act as “danger signals” detected by inflammasomes, multi-protein complexes employed by the innate immune system that proteolytically mature pro-IL-1β and pro-IL-18 into bioactive cytokines that induce sterile inflammation (Goldberg and Dixit, 2015). In addition to their beneficial roles, IL-1β and IL-18 also contribute to functional decline and disease during aging (Goldberg and Dixit, 2015; Xia et al., 2016). Accumulating evidence suggests that low grade chronic inflammation contributes to age-related functional decline. Several studies in mice have shown that disruption of the NLR Family Pyrin Domain Containing 3 (NLRP3) inflammasome reduces inflammation, prolongs the healthy lifespan and delays the aging process (Youm et al., 2013; Marín-Aguilar et al., 2020). NLRP3 is a sensor of molecules released by damaged cells (Danger Associated Molecular Patterns or DAMPs) and cellular stressors, most of which increase and accumulate with age (Franceschi and Campisi, 2014). When NLRP3 recognizes DAMPs, it engages the adaptor protein ASC (apoptosis-associated speck-like protein containing a CARD), which is present in other inflammasomes (e.g., AIM2), and the complex then recruits and matures the protease Caspase 1. This activated inflammasome then processes IL-1β and IL-18 precursors, which leads to secretion of the pro-inflammatory cytokines IL-1β and IL-18 (Goldberg and Dixit, 2015). Similar to *Nlrp3*^−/−^ mice, ASC-deficient mice display dramatically reduced IL-18 and IL-1β levels in serum, and slowed functional aging (Youm et al., 2013). These studies strongly support a link between NLRP3 inflammasome-dependent inflammation and age-related functional decline.

The factors responsible for diminished fertility in older women are likely to be multifactorial and have not been fully elucidated. Intriguingly, a recent study showed that ovaries from reproductively old mice (equivalent to women aged 38–45) are fibrotic with characteristics of chronic inflammation, including the expression of inflammatory genes and proteins (Briley et al., 2016). Inflammasome complexes have been predominately studied in innate immune cells, including macrophages, monocytes, and neutrophils (Goldberg and Dixit, 2015). However, recent studies indicate that epithelial cells of different tissues can express active inflammasome complexes (Santana et al., 2016). In addition, the expression of both NLRP3 protein and the adaptor molecule ASC have been previously detected in the C57BL/6 mouse ovary (Zhang et al., 2019). ASC was detected in oocytes, theca cells, and stroma, whereas NLRP3 was localized to the oocytes, theca cells, follicular fluid, and stroma (Zhang et al., 2019). Furthermore, a recent publication has shown an age-associated accumulation of DAMPs (i.e., lipofuscin) in the ovarian tissue (Urzua et al., 2018). In this publication, Urzua et al. demonstrated an excessive accumulation of incompletely digested cell debris in the form of lipofuscin in reproductively aged mouse ovaries (Urzua et al., 2018). More recently, Rowley et al. reported that age-related DAMPs, such as low molecular weight (LMW) hyaluronan fragments, may be a potential driver of the age-associated inflammation characteristic of ovaries from reproductively old mice (Rowley et al., 2020). However, whether accumulation of LMW hyaluronan fragments occurs in advanced reproductively aged ovaries is still under investigation. Therefore, in this study, we investigated the hypothesis that chronic low-grade systemic and local inflammation mediated by the inflammasome contributes to loss of ovarian follicular reserves as females age. To do this, the systemic and intra-ovarian inflammatory phenotype were investigated, and immune cell populations and ovarian follicles were quantified in ASC- and NLRP3-deficient mice.

## Materials and Methods

### Animals

Female C57BL/6J wild-type (WT), *Asc*^−/−^ and *Nlrp3*^−/−^ mice on a C57BL/6J background were housed in a temperature-controlled high-barrier facility (Monash University ARL) with a 12 h light-dark cycle and free access to mouse chow and water. All animal procedures and experiments were compliant with the NHMRC Australian Code of Practice for the Care and Use of Animals and approved by the Monash Animal Research Platform Animal Ethics Committee. *Asc*^−/−^ and *Nlrp3*^−/−^ mice have been previously described (Kanneganti et al., 2006; Ozoren et al., 2014). Animals were aged to 2 (young sexually mature) (WT *n* = 6, *Asc*^−/−^ *n* = 6, *Nlrp3*^−/−^ *n* = 6), 6 (adult peak fertility) (WT *n* = 8, A*sc*^−/−^ *n* = 8, *Nlrp3*^−/−^ *n* = 8), 12 (reproductively old) (WT *n* = 11, A*sc*^−/−^ *n* = 13, *Nlrp3*^−/−^ *n* = 11), and 18 months (extreme reproductively aged) (WT *n* = 8, A*sc*^−/−^ *n* = 6, *Nlrp3*^−/−^ *n* = 4), and ovaries and blood were collected. Age of mice was chosen based on previous publications (Finch et al., 1984; Kevenaar et al., 2006; Finch, 2014; Liew et al., 2014). Serum was obtained by centrifugation at 5,000 rpm for 5 min, then transferred to a sterile Eppendorf tube, and stored at −80°C. One ovary from each mouse was either fixed in Bouin's solution, frozen at −80°C or directly used for Flow Cytometry. For flow cytometric study, stage of estrous cycle was determined by vaginal cytology immediately after mice being sacrificed at 9 months of age and recorded in Supplementary Table 1. Vaginal smears were stained with Rapid Diff Stain Kit (Australian Biostain, ARD.K) and classified based on the different cell types present as previously described (Ora et al., 2015). For Bouin's, tissues were fixed overnight at 4°C, and then washed three times with 70% ethanol.

### Gene Expression

One frozen ovary per mouse was homogenized using a Retsch mixer Mill MM 400. Total RNA was extracted using RNeasy Mini kit (Qiagen, 74104) followed by DNase I treatment (Qiagen, 79254) to ensure the complete removal of any DNA contamination. RNA concentration and purity were measured using the NanoDrop 2000 spectrophotometer (ThermoFisher Scientific). Five hundred nanogram of RNA were subsequently reverse transcribed to cDNA using a SuperScript III First-Strand synthesis kit (ThermoFisher Scientific, 18080051). A Bio-Rad CFX384 machine was used for quantitative real-time PCR using the 2x QuantiNova SYBR Green PCR Master Mix as described by the manufacturer (Qiagen, 208052). GAPDH was used as the endogenous control. Results are expressed as fold change in expression compared to 2-month-old WT mice and were calculated using the 2^−Δ*ΔCt*^ method. The thermal cycling conditions used for 2x QuantiNova SYBR Green PCR Master Mix were 2 min at 95°C, followed by 40 cycles of 5 s at 95°C and 10 s at 60°C. The specificity of the process was controlled by Melting Curve analyses. The primers used in this study are detailed in Supplementary Table 2.

### Histology

Bouin's-fixed ovaries were embedded and processed in Glycol Methacrylate (GMA) resin and serially sectioned at 20 μm. Every third resin section was collected, stained with Periodic Acid-Schiff (PAS) and counterstained with hematoxylin for follicle quantification and the assessment of ovarian volume.

### Quantification of Ovarian Follicles

Primordial and primary follicle numbers were estimated using unbiased stereology, while number of growing and atretic follicles and corpora lutea numbers were quantified by direct counting under a Nikon light microscope, as previously described (Myers et al., 2004; Sarma et al., 2020; Winship et al., 2020).

### Ovarian Volume

Ovarian volume was assessed using the Cavalieri Estimator function on the Stereo Investigator version 2018.2.2 software on an Olympus BX50 microscope with a 10x objective. Briefly, a point-counting grid with a density of 200 μm was used to estimate the area of every sixth section through the ovary. Final ovarian volume was calculated using the following equation: $\vee_{ov}^{\;}=\;a\;\left(p\right)dt\overset{n}{\underset{i=1}{\sum }}P_{i},$ where *a* (*p*) is the area associated with each grid point, *d* is the distance between two consecutive sections, *t* is the section thickness and $\overset{n}{\underset{i=1}{\sum }}P_{i}$ is the sum of all points counted in each section.

### Quantifying Serum Anti-Müllerian Hormone

Serum anti-Müllerian hormone (AMH) concentration was determined using the commercially available AMH Gen II ELISA assay kit (Beckman Coulter, California, A79766) according to the manufacturer's instructions. AMH levels were measured for serum samples of 12-month-old WT (*n* = 6), *Asc*^−/−^ (*n* = 6), and *Nlrp3*^−/−^ mice (*n* = 6). Serum samples were assayed in duplicates and the absorbance was measured using the CLARIOstar microplate reader (BMG Labtechset, Ortenberg, Germany).

### Cytokine Measurement

Serum IL-6, TNF-α, and IL-18 concentrations were measured by a specific murine ELISA kit [Biolegend, 431307 (IL-6) and 430907 (TNF-α) and Abcam, ab216165 (IL-18)] according to the manufacturer's instructions. Serum samples were assayed in duplicates and absorbance read using the CLARIOstar microplate reader (BMG Labtechset, Ortenberg, Germany).

### Flow Cytometry

Fresh ovaries from 9-month-old WT (*n* = 5) and *Asc*^−/−^ mice (*n* = 3) were digested in a 37°C shaker at 120 rpm in digestion buffer (Dulbecco's phosphate-buffered saline (DPBS, ThermoFisher Scientific, 14190250) with 0.4% Collagenase IV (Sigma-Aldrich, AC5138), 0.1% Deoxyribonuclease I (Sigma-Aldrich, DN25), 0.2% Dispase II (Sigma-Aldrich, D4693), and 0.2% Hyaluronidase (Sigma-Aldrich, H3506) for 30 min. 1 ml of neutralization buffer (DPBS containing 20% dialyzed FBS (Assay Matrix, ASFBS-U) and 5 mM EDTA) was added to the buffer to stop the digestion. Cell pellet was collected by centrifugation and resuspended in FACS buffer [DPBS containing 0.5% Bovine Serum Albumin (BSA, Sigma-Aldrich, A9418) and 5 mM EDTA]. Cell suspension was then filtrated and counted.

For staining, ovarian cell suspensions were blocked with an Fc blocking agent for 10 min. Isolated cells were then incubated with the antibodies of interest for 30 min and stained with Live/Dead Viability Stain 700 (FVS700, 1:2,000, BD Biosciences, 564997) for 10 min. Finally, stained cells were transferred to round bottom polypropylene FACS tubes (Falcon). All staining protocol was carried out at 4°C and FACS buffer was used as the diluent for each stain unless indicated otherwise. Data was collected on a BD LSRFortessa X-20 cell analyzer (BD Biosciences, NSW, Australia) and analyzed using FlowJo version 10 (Tree Star Inc., Oregon, USA). Antibodies and stains used in this study are detailed in Supplementary Table 3.

### Stimulation of Ovulation and Oocyte Collection

Ovulation was induced in 12-month-old WT (*n* = 3), *Asc*^−/−^ (*n* = 5), and *Nlrp3*^−/−^ mice (*n* = 3) by intraperitoneal injection with pregnant mare serum gonadotropin (15 IU PMSG; Intervet) followed 44–48 h later by human chorionic gonadotropin (15 IU hCG; Intervet). After 12–16 h, cumulus oocyte complexes (COC) were collected from the oviduct and fully mature MII stage oocytes were exposed by digestion in M2 media with 0.3% hyaluronidase (Sigma-Aldrich).

### Statistical Analysis

Data are presented as mean ± standard error of the mean (SEM). Statistical analyses were analyzed using GraphPad Prism version 8 (GraphPad Software, California, USA). Data normality was tested using Shapiro-Wilk test. Within each age group, data from *Asc*^−/−^ or *Nlrp3*^−/−^ mice were compared with WT. Normally distributed data were analyzed using unpaired Student's *t*-test. Not normally distributed data were analyzed by Mann-Whitney test. Differences were considered significant when *p* < 0.05.

## Results

### Pro-inflammatory Cytokine Levels Are Reduced in the Serum of Aging *Asc^−/−^* Mice

**Figure 1:**
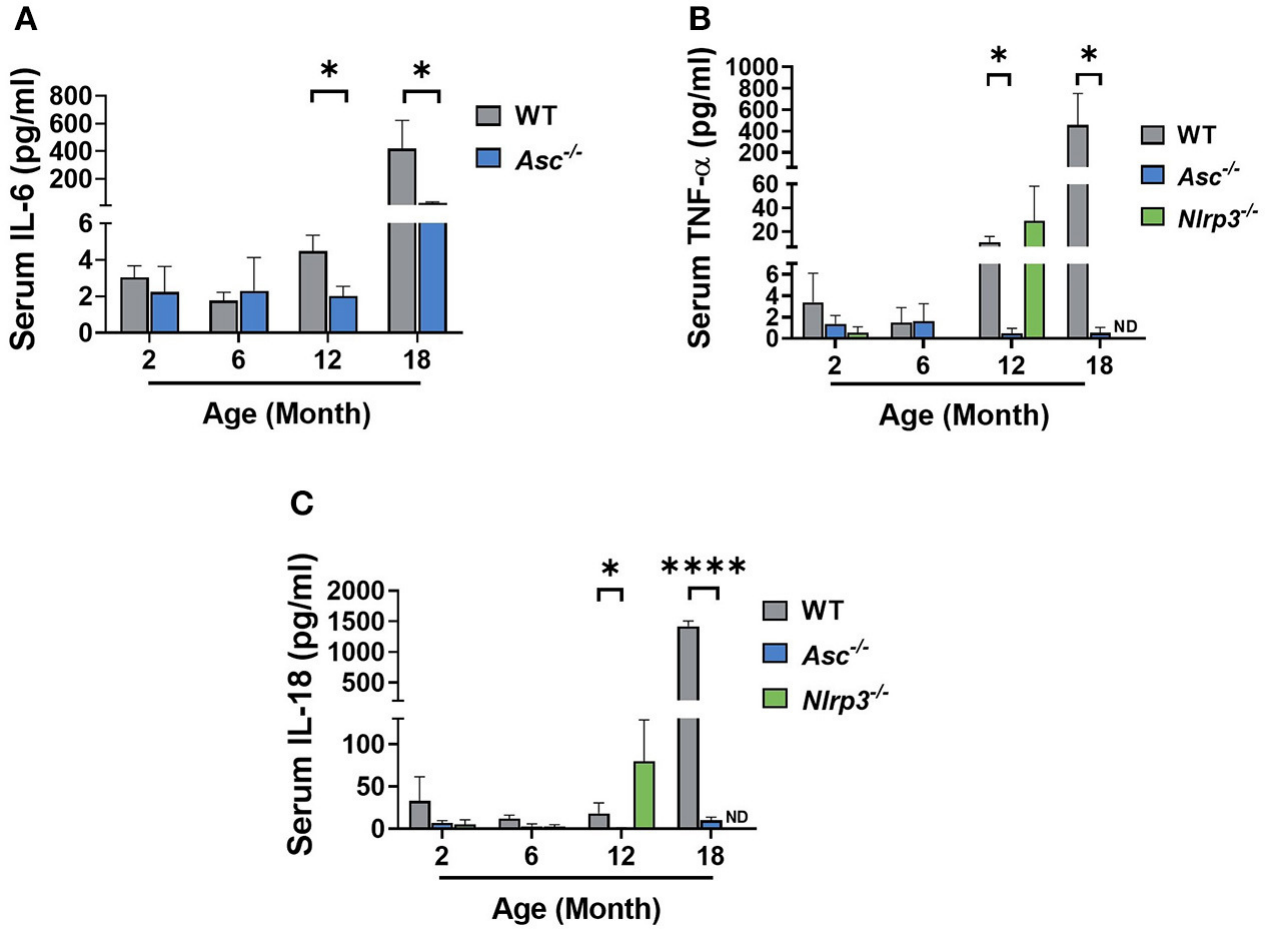
Pro-inflammatory cytokine levels are reduced in the serum of aging *Asc*^−/−^ mice. Serum IL-6 **(A)** concentration measured in 2, 6, 12, and 18-month-old WT and *Asc*^−/−^ mice. Circulating levels of TNF-α **(B)** and IL-18 **(C)** determined in 2, 6, 12, and 18-month-old WT, *Asc*^−/−^, and *Nlrp3*^−/−^ mice. *n* = 4–6 per cohort. ND, Not Determined. Data are presented as mean ± SEM. For each age group, comparisons were made with WT using Student's *t*-test **(A,C)** or Mann-Whitney test **(A–C)** (*p < 0.05, ****p < 0.0001).

The NLRP3 inflammasome is a major driver of age-related sterile inflammation in multiple organs (Youm et al., 2013; Goldberg and Dixit, 2015). To determine if loss of ASC or NLRP3 reduces systemic inflammation, pro-inflammatory cytokines were measured in serum from 2, 6, and 12-month-old WT, *Asc*^−/−^, and *Nlrp3*^−/−^ mice and 18-month-old WT and *Asc*^−/−^ mice (Figure 1). IL-6, TNF-α, and IL-18 all increased with age in the serum of WT mice. However, while circulating levels of IL-6, TNF-α, and IL-18 were similar between genotypes at 2 and 6 months, their expression was significantly lower in *Asc*^−/−^ mice than WT mice at 12 and 18 months (Figures 1A–C). In contrast to *Asc*^−/−^ mice, TNF-α and IL-18 were not significantly different in *Nlrp3*^−/−^ from age-matched WT mice at 12 months (*p* > 0.05) (Figures 1B,C). For *Nlrp3*^−/−^ mice, the concentration of TNF-α and IL-18 in serum at 18 months, and IL-6 at all ages, was not determined due to lack of serum availability. These data indicate that deletion of adaptor protein ASC, but not NLRP3, attenuates the levels of systemic inflammatory cytokines observed in mice during reproductive aging.

### NLRP3 Inflammasome

**Figure 2:**
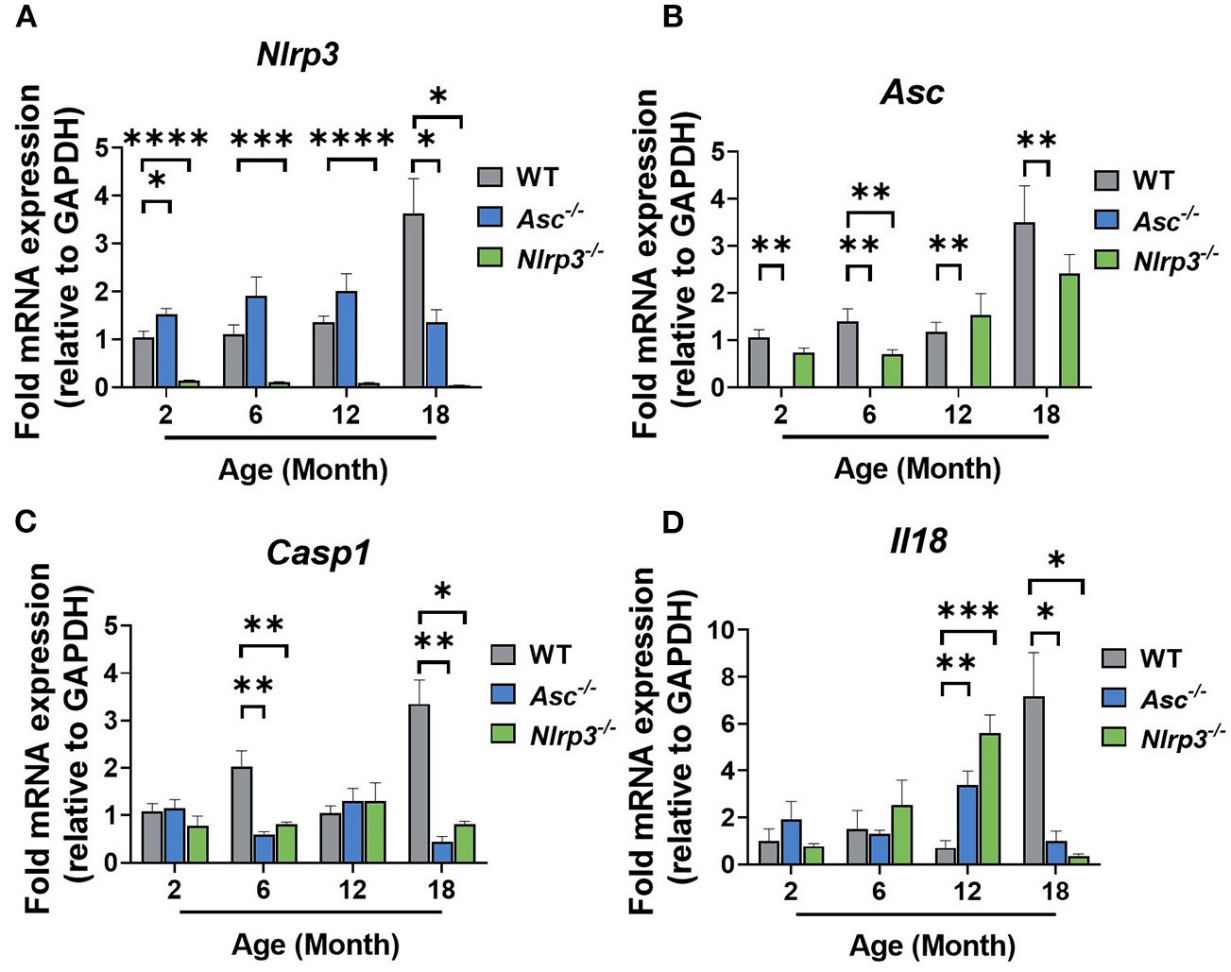
Gene expression levels of inflammasome-related genes Nlrp3 **(A)** and Asc **(B)**, caspase 1 **(C)** and inflammasome-associated cytokine Il18 **(D)** in ovaries from 2, 6, 12, and 18-month-old WT, *Asc*^−/−^, and *Nlrp3*^−/−^ mice. *n* = 3–6 per cohort. Data are presented as mean ± SEM. For each age group, comparisons were made with WT using Student's *t*-test **(A–D)** or Mann-Whitney test **(D)** (*p < 0.05, **p < 0.01, ***p < 0.001, ****p < 0.0001).

To examine the intra-ovarian expression profile of the NLRP3 inflammasome, the mRNA expression levels of Nlrp3 and Asc were determined at 2, 6, 12, and 18-months of age (Figures 2A,B). As expected, Nlrp3 and Asc genes were not expressed in *Nlrp3*^−/−^ and *Asc*^−/−^ ovaries at any age, respectively (Figures 2A,B). Nlrp3 expression in 2, 6, and 12-month-old *Asc*^−/−^ mice was similar to WT mice and decreased significantly at 18 months of age (Figure 2A). Asc mRNA levels in *Nlrp3*^−/−^ mice only showed a significant decrease at 6 months compared to WT mice (Figure 2B).

To further investigate the effect of inflammasome ablation on downstream factors of NLRP3 inflammasome, caspase-1 and IL-18, mRNA expression levels of caspase 1 and Il18 were analyzed in ovaries from WT, *Asc*^−/−^, and *Nlrp3*^−/−^ ovaries (Figures 2C,D). Casp1 mRNA expression was significantly decreased at 6 and 18 months of age in *Asc*^−/−^ and *Nlrp3*^−/−^ mice compared to WT mice (Figure 2C). mRNA levels of Il18 were increased in 12-month-old *Asc*^−/−^ and *Nlrp3*^−/−^ mice and significantly decreased at 18 months of age compared to WT mice (Figure 2D).

Taken together, corresponding with Casp1, Nlrp3, Il1b, and Il18 as NF-κB-dependent genes, reduction of these components and substrates of the inflammasome is consistent with a reduction in age-related systemic inflammation.

### The Expression of Pro-inflammatory Genes Is Reduced in Ovaries From Aging *Asc^−/−^* Mice

**Figure 3:**
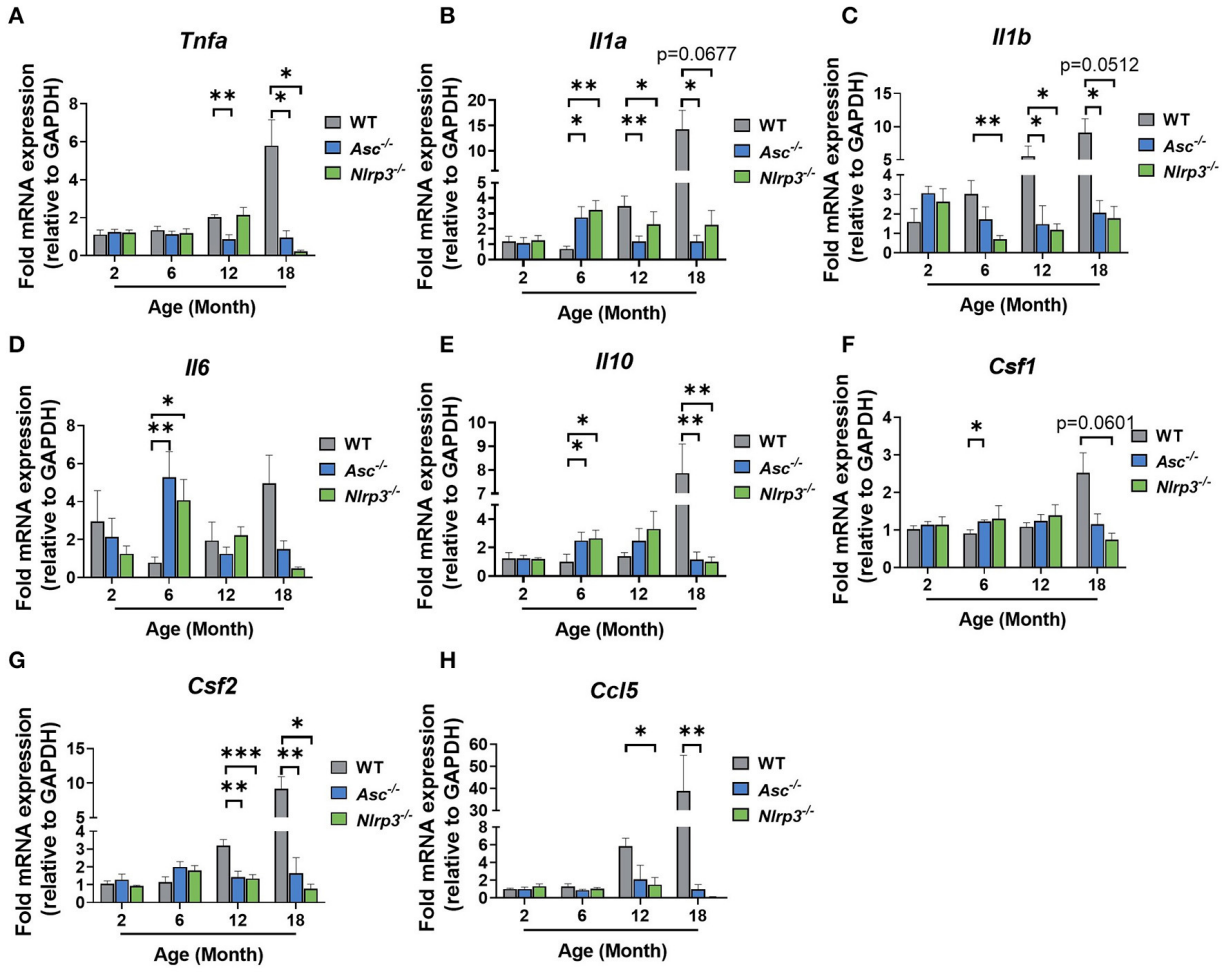
The expression of pro-inflammatory genes is reduced in ovaries from aging *Asc*^−/−^ mice. Gene expression levels of Tnfa **(A)**, Il1a **(B)**, Il1b **(C)**, Il6 **(D)**, Il10 **(E)**, Csf1 **(F)**, Csf2 **(G)**, and Ccl5 **(H)** in ovaries from 2, 6, 12, and 18-month-old WT, *Asc*^−/−^, and *Nlrp3*^−/−^ mice. *n* = 3–6 per cohort. All data are presented as mean ± SEM. For each age group, comparisons were made with WT using Student's *t*-test **(A–H)** or Mann-Whitney test **(A–H)** (*p < 0.05, **p < 0.01, ***p < 0.001).

We next investigated the effect of ASC or NLRP3 deletion on intra-ovarian inflammation. mRNA levels of major pro-inflammatory genes Tnfa, Il1a, Il1b, and Il6 were examined in 2, 6, 12, and 18-month-old WT, *Asc*^−/−^, and *Nlrp3*^−/−^ ovaries (Figures 3A–D).

In general, Tnfa, Il1a, Il1b mRNA levels tended to increase gradually with age in WT ovaries, with significantly higher levels observed at 18 months than 2 months (Figures 3A–D). In contrast, the expression of Tnfa, Il1a, Il1b did not increase with age in ovaries from *Asc*^−/−^ mice, and mRNA levels were significantly lower than in WT ovaries at 12 and 18 months (Figures 3A–C). Similarly, in ovaries from *Nlrp3*^−/−^ mice, Il1a and Il1b were significantly decreased relative to WT mice at 12 months of age, and Tnfa was decreased at 18 months (Figures 3A–C). Unexpectedly, significantly higher expression levels of Il6 were observed in *Asc*^−/−^ and *Nlrp3*^−/−^ ovaries compared to WT at 6 months of age, but this increase was transient and not observed at any other age (Figure 3D).

Expression levels of anti-inflammatory gene Il10 were also measured (Figure 3E). A significant increase in the mRNA levels was observed in ovaries from *Asc*^−/−^ and *Nlrp3*^−/−^ mice relative to WT mice at 6 months, while at 18 months, significantly lower expression levels of Il10 was observed in knock-out mice compared to ovaries from WT mice (Figure 3E).

Next, the expression pattern of Csf1 and Csf2 were examined because of their involvement in the regulation of macrophages and granulocytes (Figures 3F,G). While Csf1 mRNA showed a small increase at 6 months in *Asc*^−/−^ mice compared to WT, no other changes were evident (Figure 3F). However, Csf2 mRNA expression was significantly lower in ovaries from 12 and 18-month-old *Asc*^−/−^ and *Nlrp3*^−/−^ mice compared to WT ovaries (Figure 3G). Finally, mRNA levels of Ccl5, a chemokine that mediates recruitment of leukocytes to inflammatory sites, was also measured (Figure 3H). Gene expression was significantly decreased in ovaries from 12 and 18-month-old *Asc*^−/−^ and *Nlrp3*^−/−^ mice compared to WT (Figure 3H).

Taken together, these data indicate that mRNA expression markers of age-associated inflammatory response in aged *Asc*^−/−^ and *Nlrp3*^−/−^ ovaries are reduced compared to WT ovaries. Expression levels of inflammatory cytokines Tnfa, Il1a, Il1b, and Csf2, as well as Ccl5 chemokine, were significantly lower in reproductively old inflammasome-deficient mice, suggesting that migration to inflammatory sites and subsequent stimulation of immune cells, is impaired in the ovarian tissue due to inflammasome ablation.

### Immune Cell Populations in the Mouse Ovary Were Significantly Decreased by ASC Ablation

Since our results indicated that systemic and intra-ovarian pro-inflammatory levels were lower in *Asc*^−/−^ mice than WT mice during reproductive aging, we next examined local immune cell populations in 9-month-old *Asc*^−/−^ ovaries. The gating strategy used in this study was conducted as described in Supplementary Figure 1. In brief, ovarian cells were first gated for singlets (FSC-H vs. FSC-A) and lymphoid/myeloid populations (SSC-A vs. FSC-A). CD11b, TCRβ, NK1.1, and CD19 expression were used to identify different immune cell populations, analyzing only live cells. NK cells were gated as NK1.1+TCRβ- and B cells as CD19+. T cells were identified based on TCR expression (TCRβ+) and either CD4 or CD8. Finally, F4/80 surface marker was used to identify macrophages within the CD11b+ population (F4/80+CD11b+).

**Figure 4:**
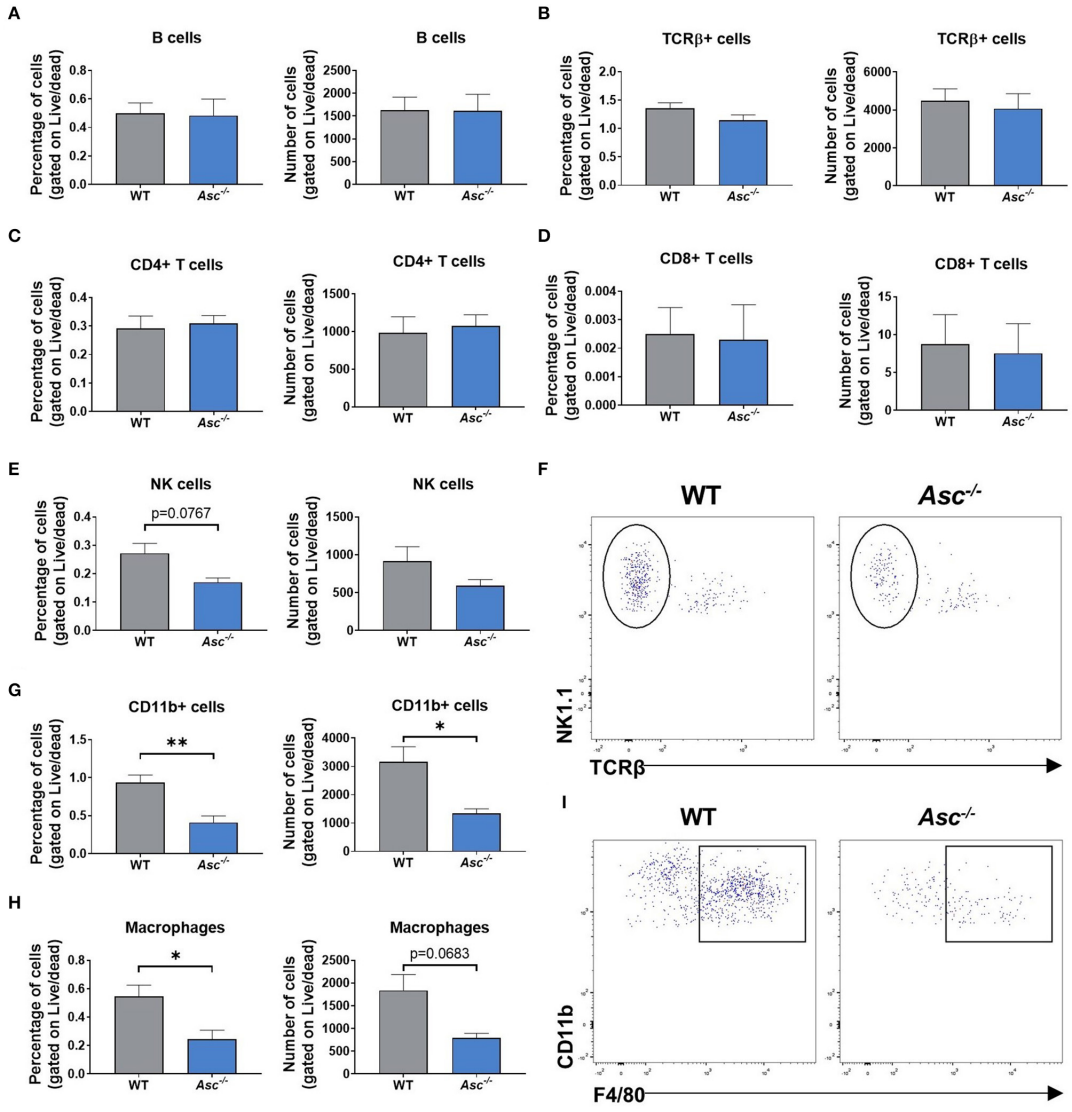
Immune cell populations in the mouse ovary were significantly decreased by ASC ablation. Percentage and number of B cells **(A)**, T cells **(B)**, CD4+ **(C)**, and CD8+ T cells **(D)**, NK1.1+TCRβ- NK cells **(E)**, CD11b+ cells **(G)**, and F4/80+CD11b+ macrophages **(H)** present in ovaries from 9-month-old WT and *Asc*^−/−^ mice. *n* = 3–5 per cohort. Representative dot plots of NK1.1+TCRβ- NK cells **(F)** and F4/80+CD11b+ macrophages **(I)** in ovaries from 9-month-old WT and *Asc*^−/−^ mice. Data are presented as mean ± SEM. For each age group, comparisons were made with WT using Student's *t*-test **(A–H)** or Mann-Whitney test **(D)** (*p < 0.05, **p < 0.01).

Lymphoid cell populations, including B and T cells, did not show any significant change in *Asc*^−/−^ ovaries compared to WT, either in the percentage or number of cells (*p* > 0.05) (Figures 4A–D). The percentage of NK cells showed a trend toward a decrease in ovaries from *Asc*^−/−^ mice compared to WT mice (*p* = 0.0767) (Figures 4E,F).

Importantly, significant differences were observed in myeloid populations. The proportion and number of ovarian cells comprising the CD11b+ population was significantly decreased in *Asc*^−/−^ ovaries compared to WT (Figure 4G). Consistent with CD11b+ cell population data, a significant decrease was also observed in the percentage of F4/80+CD11b+ macrophages in ovaries from *Asc*^−/−^ mice relative to WT mice, while the number of macrophages showed a trend toward a decrease in *Asc*^−/−^ ovaries compared to WT (*p* = 0.0683) (Figures 4H,I). Thus, our findings suggest that ablation of ASC attenuate macrophage presence in the mouse ovary, which may lead to the reduction of expression levels of inflammatory genes like Tnfa, Il1a, and Ccl5.

### Loss of ASC or NLRP3 Significantly Preserves the Primordial Follicle Pool in Reproductively Old Mice

**Figure 5:**
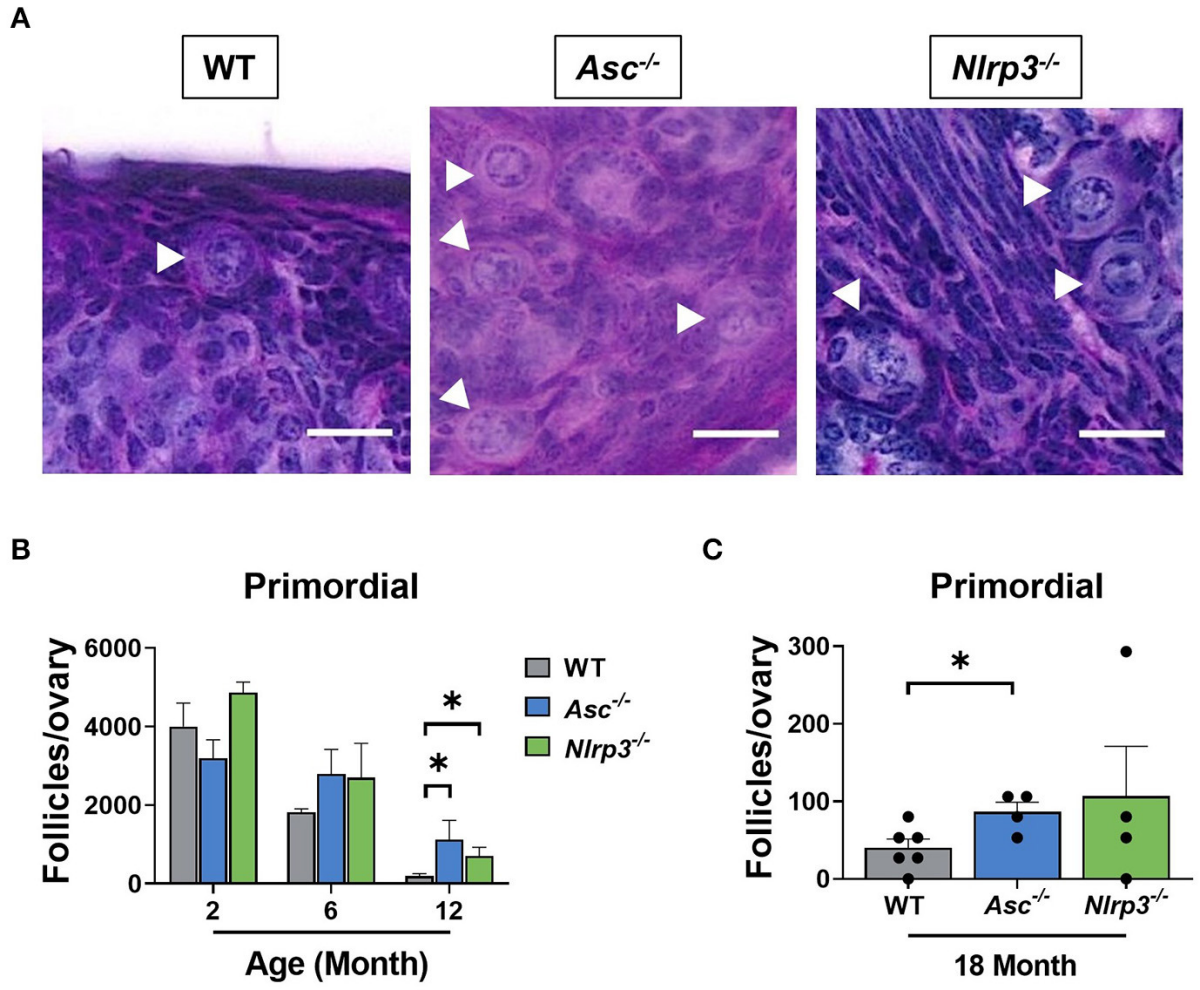
Loss of ASC or NLRP3 significantly preserves the primordial follicle pool in reproductively old mice. **(A)** Representative images of PAS-stained ovaries of 12-month-old WT, *Asc*^−/−^, and *Nlrp3*^−/−^ mice. White head arrows indicate primordial follicles. Scale bars are 20 μm. Primordial **(B)** follicle numbers in ovaries from 2, 6, and 12-month-old WT, *Asc*^−/−^, and *Nlrp3*^−/−^ mice. *n* = 3–6 per cohort. Primordial **(C)** follicle numbers in ovaries from 18-month-old WT, *Asc*^−/−^, and *Nlrp3*^−/−^ mice. *n* = 4–6 per cohort. Each dot represents one animal. Data are presented as mean ± SEM. For each age group, comparisons were made with WT using Student's *t*-test **(A,B)** or Mann-Whitney test **(A)** (*p < 0.05).

The ovarian reserve constitutes a finite reservoir of primordial follicles in the ovaries that determines the length of the female fertile lifespan (Findlay et al., 2015, 2018). Some studies have suggested that inflammatory cytokines, such as TNF-α, have an impact on primordial follicle survival (Morrison and Marcinkiewicz, 2002; Greenfeld et al., 2007). Therefore, primordial follicles were examined in ovaries from 2, 6, 12, and 18-month-old WT, *Asc*^−/−^, and *Nlrp3*^−/−^ mice in order to investigate the contribution of the inflammasome-dependent inflammation to the age-related follicle depletion (Figure 5). At 2 and 6 months of age, primordial follicle numbers in ovaries from *Asc*^−/−^ and *Nlrp3*^−/−^ mice were not significantly different from WT ovaries. These data suggest that reproductively young inflammasome-deficient mice have normal primordial follicle endowment. Critically, ovaries from 12-month-old *Asc*^−/−^ and *Nlrp3*^−/−^ mice contained significantly more primordial follicles than WT mice, consistent with retention of the ovarian reserve during reproductive aging (Figure 5B). Even at 18 months of age, when the pool of primordial follicles was severely depleted in WT ovaries, *Asc*^−/−^ mice still retain elevated primordial follicles (Figure 5C). A similar trend was observed for *Nlrp3*^−/−^ mice at 18 months, though the difference with WT was not statistically significant (Figure 5C).

### Loss of ASC or NLRP3 Significantly Preserves the Growing Follicle Pool in Reproductively Old Mice

**Figure 6:**
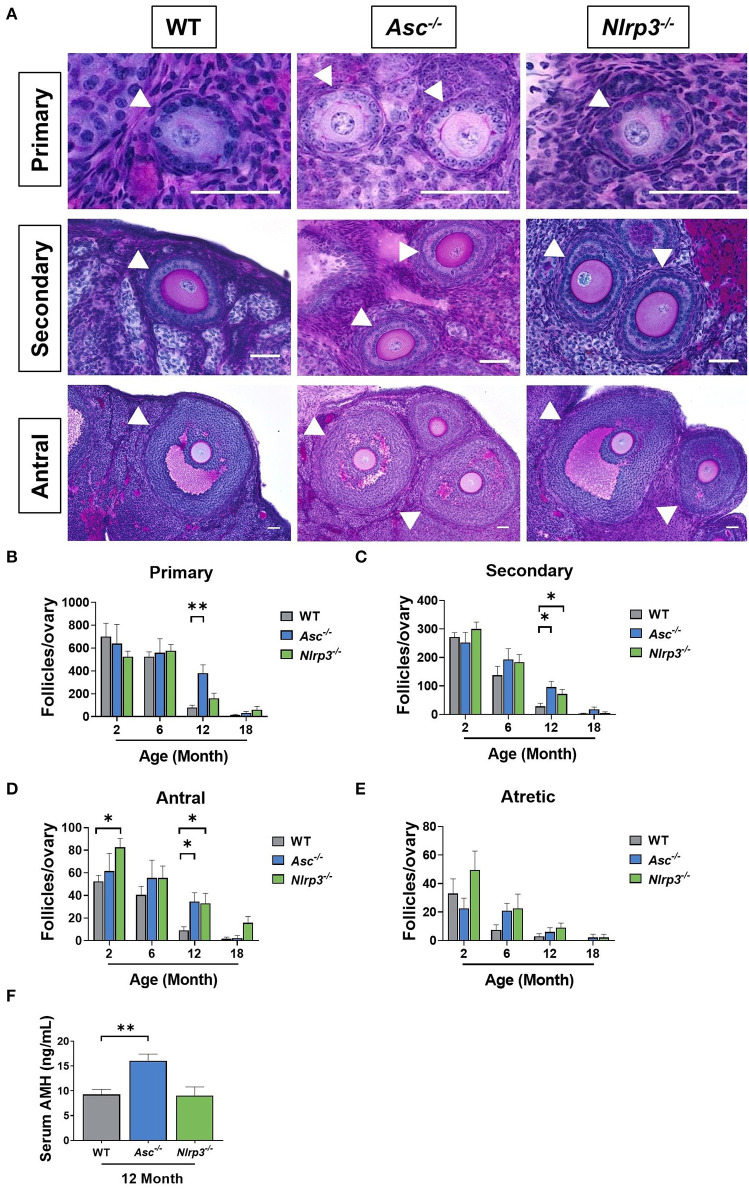
Loss of ASC or NLRP3 significantly preserves the growing follicle pool in reproductively old mice. **(A)** Representative images of PAS-stained ovarian sections showing primary, secondary and antral follicles of 12-month-old WT, *Asc*^−/−^, and *Nlrp3*^−/−^ mice. Scale bars are 50 μm. Primary, secondary and antral follicles are indicated by white head arrows. Primary **(B)**, secondary **(C)**, antral **(D)**, and atretic **(E)** follicle numbers in ovaries from 2, 6, 12, and 18-month-old WT, *Asc*^−/−^, and *Nlrp3*^−/−^ mice. *n* = 4–6 per cohort. Serum AMH **(F)** concentration in 12-month-old WT, *Asc*^−/−^, and *Nlrp3*^−/−^ mice. *n* = 6 per cohort. All data are presented as mean ± SEM. For each age group, comparisons were made with WT using Student's *t*-test **(B–F)** or Mann-Whitney test **(B–F)** (*p < 0.05, **p < 0.01).

The number of healthy and atretic growing follicles were determined in ovaries from 2, 6, 12, and 18-month-old WT, *Asc*^−/−^, and *Nlrp3*^−/−^ mice (Figures 6A–E). Consistent with our findings for primordial follicles, the number of primary, secondary and antral follicles was similar between WT and *Asc*^−/−^ ovaries at 2 and 6 months. However, a significant increase in the number of growing follicles at each of these three stages was observed in 12-month-old *Asc*^−/−^ ovaries compared to WT ovaries (Figures 6A–D). Secondary and antral follicle numbers were also significantly increased in *Nlrp3*^−/−^ mice at 12 months of age compared to WT mice (Figures 6C,D). By 18 months of age growing follicle numbers were not statistically significantly different between genotypes, however, there was a trend toward more antral follicles in *Nlrp3*^−/−^ mice. There were no significant changes in the atretic follicle number between groups (*p* > 0.05) (Figure 6E).

AMH is produced by granulosa cells in growing follicles, with the highest production from secondary and early antral follicles and is often used as a clinical marker of the ovarian reserve (Kevenaar et al., 2006). To investigate the impact of losing ASC or NLRP3 function on AMH levels, serum was collected from 12-month-old *Asc*^−/−^ and *Nlrp3*^−/−^ mice. A significant increase in the serum AMH concentration was observed in 12-month-old *Asc*^−/−^ mice compared to age-matched WT mice (Figure 6F). This finding is consistent with observations that primary, secondary and antral follicle numbers were significantly higher in 12-month-old *Asc*^−/−^ relative to WT mice (Figures 6B–D). There were no differences in serum AMH levels between 12-month-old WT and *Nlrp3*^−/−^ mice (*p* > 0.05) (Figure 6F), despite increased numbers of secondary and antral follicles.

Overall, these results demonstrate that deletion of NLRP3 or ASC preserves follicle numbers during aging in mice.

### Loss of ASC or NLRP3 Increases the Number of Corpora Lutea in Reproductively Aged Mice

**Figure 7:**
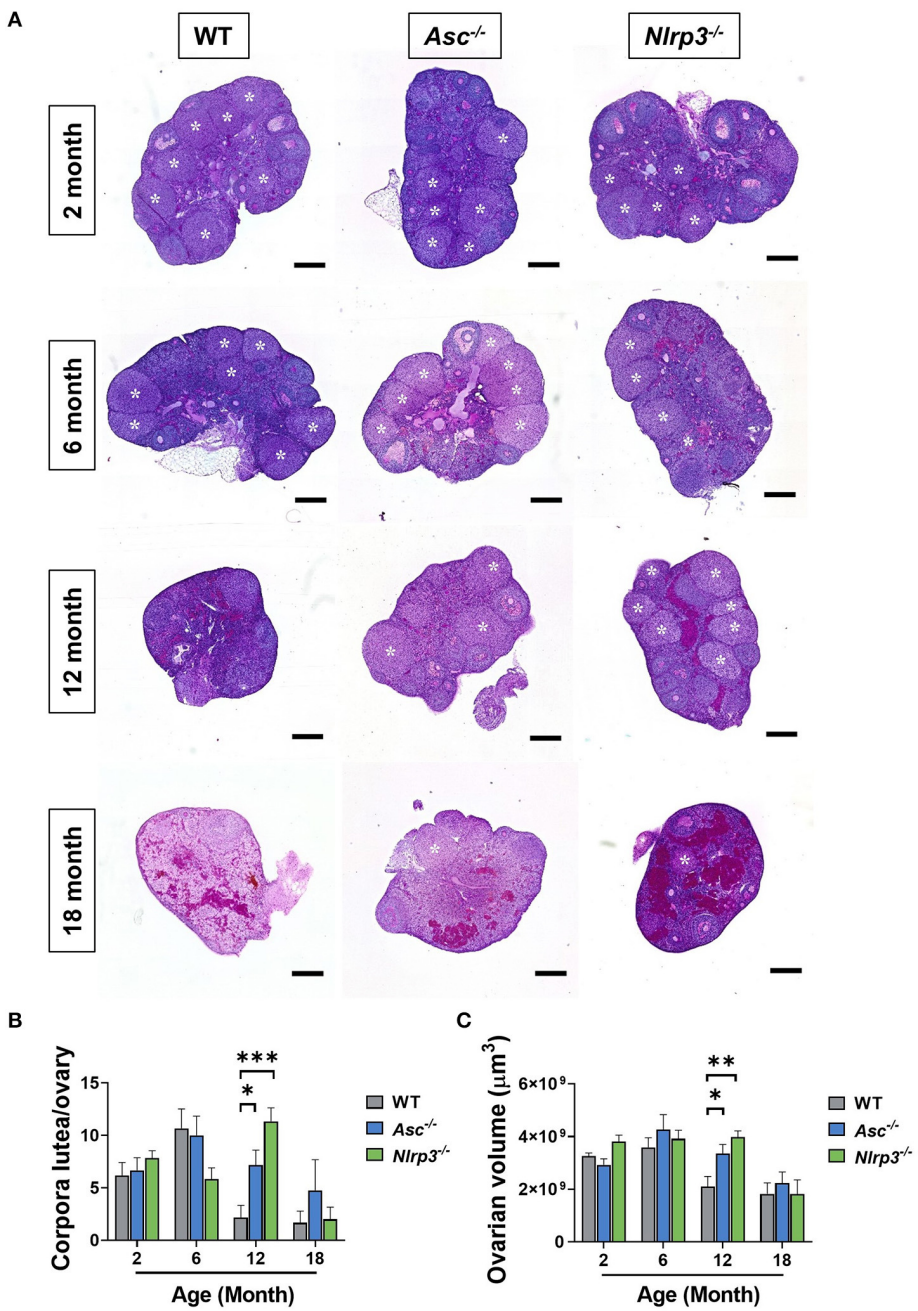
Loss of ASC or NLRP3 increases the number of corpora lutea in reproductively aged mice. **(A)** Representative images of whole ovarian sections of 2, 6, 12, and 18-month-old WT, *Asc*^−/−^, and *Nlrp3*^−/−^ mice. White asterisks indicate corpora lutea. Scale bars are 400 μm. Corpora lutea numbers **(B)** in ovaries from 2, 6, 12, and 18-month-old WT, *Asc*^−/−^, and *Nlrp3*^−/−^ mice. Ovarian volume **(C)** for 2, 6, 12, and 18-month-old WT, *Asc*^−/−^, and *Nlrp3*^−/−^ mice. *n* = 4–6 per cohort. All data are presented as mean ± SEM. For each age group, comparisons were made with WT using Student's *t*-test (*p < 0.05, **p < 0.01, ***p < 0.001).

Corpora lutea are an indirect marker of recent ovulation. To determine if loss of ASC or NLRP3 function impacts ovulation, corpora lutea were quantified in 2, 6, 12, and 18-month-old WT, *Asc*^−/−^, and *Nlrp3*^−/−^ mice (Figures 7A,B). Corpora lutea number was similar between genotypes in reproductively young mice. Older WT mice had few corpora lutea, but a striking increase was observed in ovaries from reproductively aged 12-month-old *Asc*^−/−^ and *Nlrp3*^−/−^ mice relative to age-matched WT mice (Figures 7A,B). In particular, it was noted that the number of corpora lutea in 12-month-old *Nlrp3*^−/−^ mice was similar to that observed in 6-month-old WT mice.

Corpora lutea typically grow from 200 to 750 μm in mice (Mircea et al., 2009), making a large contribution to the total ovarian volume. Ovarian volume was assessed in 2, 6, 12, and 18-month-old WT, *Asc*^−/−^, and *Nlrp3*^−/−^ mice (Figure 7C). Ovaries from 12-month-old WT mice were significantly smaller compared to age-matched A*sc*^−/−^ and *Nlrp3*^−/−^ ovaries (Figure 7C), consistent with corpora lutea data (Figure 7B). By 18 months of age, there was no difference in corpora lutea numbers or volume between *Asc*^−/−^ or *Nlrp3*^−/−^ and WT mice (*p* > 0.05), consistent with the end of fertility at advanced reproductive age (Figure 7B).

These data suggest that ovaries from both *Asc*^−/−^ and *Nlrp3*^−/−^ mice remain functionally active for longer than WT mice, and indicate that the reproductive lifespan may be prolonged in these animals. To investigate ovulation rate in more detail, we hormonally stimulated 12-month-old mice using standard superovulation protocols and collected oocytes from the oviduct. Interestingly, there was no significant difference in the total number or MII-stage ovulated oocytes collected from *Asc*^−/−^, *Nlrp3*^−/−^, and WT mice (Supplementary Figure 2). Indeed, the ovulated oocytes that were not MII were mostly fragmented oocytes. Thus, even though natural ovulation might be improved in inflammasome-deficient mice during aging (as indicated by increased follicles and corpora lutea), artificial induction of ovulation did not result in the maturation of more oocytes in *Asc*^−/−^ or *Nlrp3*^−/−^ female mice.

## Discussion

Recent studies have suggested that the NLRP3 inflammasome has a major influence on inflammaging, which is proposed to underlie a range of pathologies associated with the normal aging process (Goldberg and Dixit, 2015; Latz and Duewell, 2018). In this study, we investigated the hypothesis that the inflammasome, and the NLRP3 inflammasome in particular, contributes to age-related follicle depletion. We found that deletion of the inflammasome adaptor protein ASC in mice lowered serum pro-inflammatory cytokine levels, reduced the intra-ovarian expression of pro-inflammatory cytokines and myeloid cell populations, and attenuated the age-related depletion of follicles at all stages of development. Similarly, loss of NLRP3 preserved follicle numbers in older mice compared to WT controls. Loss of ASC or NLRP3 was also associated with increased CL number, which may be indicative of improved ovulatory capacity during aging. Altogether, these studies implicate the inflammasome in follicle depletion during the normal aging process and suggest that ovarian aging could be delayed by blocking the inflammasome, possibly leading to extended fertility. However, it should be noted that additional studies, including an analysis of fertility, are required to confirm the latter.

Similar to earlier studies (Youm et al., 2013), we found that systemic levels of inflammation were significantly lower in reproductively aged *Asc*^−/−^ mice than age-matched WTs. Youm et al. (2013) also reported that 23-month-old *Nlrp3*^−/−^ mice exhibited higher serum IL-18 levels than *Asc*^−/−^ mice, but significantly lower than WT mice, suggesting that *Nlrp3*^−/−^ mice also exhibit reduced inflammation during aging, although possibly to a lesser extent than in *Asc*^−/−^ mice (Youm et al., 2013). In our study, *Nlrp3*^−/−^ mice did not exhibit a decrease in the serum IL18 or TNFα, compared to age matched WT mice, but we did not examine these cytokines in the serum of mice *Nlrp3*^−/−^ mice beyond 12 months of age, which may have been too young for changes to be detected. Overall, our data are consistent with previous publications showing that disruption of the inflammasome attenuates age-associated inflammatory levels (Duewell et al., 2010; Youm et al., 2013).

In our study, inflammasome-deficient mice also exhibited reduced intra-ovarian gene expression levels of Tnfa, Il1a, and Il1b at 12 and 18 months of age, all of which have been associated with the inflammaging in other organs (Goldberg and Dixit, 2015). For example, previous studies have shown that ablation of the inflammasome significantly reduced Il1b expression in aged fat tissue and hippocampus and Tnfa levels in hippocampus and cerebral cortex from 23-month-old mice (Youm et al., 2013). Furthermore, elimination of NLRP3 inflammasome decreased levels of IL-1β cytokine in the pancreas from 12-month-old obese mice compared to age-match WT mice, as revealed by immunofluorescence (Youm et al., 2011). Interestingly, other studies have demonstrated that increased IL-6 expression is strongly associated with chronic inflammation during generalized aging (Maggio et al., 2006). Moreover, increased levels of IL-6 in the culture media from old CB6F1 mouse ovaries compared to young ovaries has been recently reported by Briley et al. (2016). However, in our study no decrease was found in IL-6 mRNA levels in ovaries from reproductively aged *Asc*^−/−^ and *Nlrp3*^−/−^ mice compared to WT mice.

Inflammasome-dependent inflammation is driven by IL-1β and IL-18 cytokines, which are able to activate the inflammatory transcription factor NF-κB (Tsuji-takayama et al., 1999; Liu et al., 2017). In addition, various pro-inflammatory genes, including Il1b and Il18, as well as inflammasome-related genes Nlrp3 and caspase 1, are transcriptionally regulated by NF-κB activation (Lee et al., 2015; Liu et al., 2017). As discussed above, in our study, Il18, Il1b, and caspase 1 mRNA expression was decreased in aged ovaries from *Asc*^−/−^ and *Nlrp3*^−/−^ mice. These data are consistent with disruption of NLRP3 inflammasome and reduced cytokine-mediated stimulation (i.e., IL-1β and TNF-α) of NF-κB, leading to a lower expression of NF-κB-dependent genes Il18, Il1b, and caspase 1. The existence of alternative pathways of IL-1β and IL-18 activation that are independent of the inflammasomes have been described (van de Veerdonk et al., 2011). For example, neutrophils are considered the principal source of proteinase 3, which processes IL-1β, during the acute bacterial and fungal infection. In the later stages of infection, caspase-1, and inflammasome would become more important for the production of mature IL-1β (van de Veerdonk et al., 2011).

The reduction in systemic and local inflammation, and retention of follicle numbers was more subtle in *Nlrp3*^−/−^ mice than *Asc*^−/−^ mice. Since ASC adaptor protein is present in other inflammasomes, such as AIM2 (Schroder and Tschopp, 2010), it is possible that other inflammasomes could also play a role in the age-associated inflammation and follicle depletion. Indeed, the AIM2 inflammasome senses host- and pathogen-associated cytosolic dsDNA, leading to caspase-1 activation and catalytic cleavage of IL-1β and IL-18 pre-forms (Kumari et al., 2020). A recent publication indicated that release of DNA after cumulative cell damage in aging cells leads to activation of cytosolic DNA sensors that trigger an inflammatory response (Lan et al., 2019). Considering these data, future experiments could investigate the possibility that accumulation of cytosolic DNA occurs in ovarian cells with age, promoting activation of AIM2 inflammasome in the aging ovary. It is also worth mentioning that recent investigations have suggested that ASC and NLRP3 exhibit an inflammasome-independent function under certain conditions. Watanabe et al. (2020) has recently reported that ASC negatively regulates GPVI signaling in platelets and enhances thrombus formation, independent of NLRP3 inflammasome and IL-1β (Watanabe et al., 2020).

Importantly, primordial follicle numbers in *Asc*^−/−^ and *Nlrp3*^−/−^ mice were similar to WT at 2 and 6 months of age, but were elevated compared to controls at 12 months. These data suggest that deletion of ASC or NLRP3 reduces the rate at which primordial follicle depletion occurs as mice age, and rules out the possibility that these mice simply entered adulthood with greater numbers of primordial follicles. Altogether, our observations suggest an association between low-grade chronic inflammation and age-related follicle depletion, as previously proposed (Cui et al., 2011; Uri-Belapolsky et al., 2014; Briley et al., 2016), as well as specifically implicating inflammasome-derived inflammation. Notably, TNF-α can promote oocyte apoptosis (Kaipia et al., 1996; Morrison and Marcinkiewicz, 2002; Greenfeld et al., 2007) *via* TNF receptor 2 located in pre-follicular oocytes, and primordial and growing follicles (Greenfeld et al., 2007; Wang et al., 2020). Thus, lower mRNA levels of Tnfa observed in ovaries from aging *Asc*^−/−^ mice could contribute to a reduction in the normal rate of primordial follicle death. Indeed, oocytes from mice lacking TNF-α exhibited lower levels of Caspase-3, leading to the larger follicle reserve observed in *Tnfa*^−/−^ mice (Cui et al., 2011). Similarly, Youm et al. indicated that obesity-induced increase in pancreatic β-cell death was reduced in *Nlrp3*^−/−^ obese mice as indicated by lower number of TUNEL-positive cells (Youm et al., 2011).

Consistent with the primordial follicle data, antral follicles and corpora lutea were increased in aged *Asc*^−/−^ and *Nlrp3*^−/−^ mice, suggesting increased ovulatory potential. Previous studies have indicated that deletion of cytokines like IL-1α or TNF-α resulted in an increased response to gonadotropins, along with an enhanced pregnancy rate and litter size (Gordon, 1999; Uri-Belapolsky et al., 2014). However, our superovulation study did not show an improved ovulation rate in inflammasome-deficient mice. Zhang et al. (2019) demonstrated that follicular expression of ASC and NLRP3 is induced by exogenous hormonal stimulation (e.g., PMSG), suggesting a possible role of NLRP3 inflammasome in the regulation of the ovulatory process (Zhang et al., 2019). A possible role for the NLRP3 inflammasome in response to hormonal priming during superovulation might explain why we saw no differences in the number of oocytes ovulated between *Asc*^−/−^, *Nlrp3*^−/−^ mice, and WT mice. Furthermore, it has been reported that treatment of female mice with supra-physiological doses of exogenous gonadotropin impacts on female reproductive organs by damaging the reproductive function and maternal environment (Park et al., 2015), which could be also affecting the outcome of the superovulation procedure. Alternatively, it is possible that the increased corpora lutea in inflammasome deficient mice could be the results of impaired corpora lutea regression. Thus, further studies are required to determine if ovulation is enhanced or corpus luteum regression underlies the observed phenotype.

Macrophages are the most abundant innate immune cells present in the ovary and are essential for ovarian function, with roles in regulating follicle growth, tissue remodeling at ovulation and formation and regression of corpus luteum (Wu et al., 2004). Interestingly, during regression of corpora lutea, the number of macrophages increases, indicating the critical role of these immune cells during luteolysis (Wu et al., 2004). Additionally, macrophages are the main source of TNF-α in the corpora lutea of different species, being this cytokine critical in the regulation of luteal angiogenesis (Lu et al., 2019). Our flow cytometry results indicate that fewer percentage of macrophages were present in *Asc*^−/−^ ovaries than in WT ovaries. Therefore, this reduced percentage of macrophages could be delaying corpora lutea regression, as previously mentioned, resulting in higher number of corpora lutea in inflammasome deficient mice.

Lower percentage of ovarian macrophages found in *Asc*^−/−^ mice is consistent with lower Ccl5 expression in the ovary, along with other inflammatory genes (e.g., Tnfa or Il1a), which may suggest that leukocyte recruitment to inflammatory sites is reduced. Zhang et al. (2020) showed that monocyte-derived macrophages were increased in aged ovaries compared to young ovaries, along with an increase in Ccl5 expression (Zhang et al., 2020). Notably, TNF-α plays a central role in activating and maintaining the inflammatory response (Parameswaran and Patial, 2010). Activated macrophages produce TNF-α *via* NF-κB transcription factor, which in turn is able to induce NF-κB activation in a self-regulating manner (Parameswaran and Patial, 2010), leading to persistent, low-grade inflammation which is characteristic of aging process (Franceschi and Campisi, 2014; Goldberg and Dixit, 2015). Within the ovary, TNF-α promotes DNA fragmentation and follicle apoptosis (Kaipia et al., 1996; Morrison and Marcinkiewicz, 2002; Greenfeld et al., 2007), thus the decreased percentage of ovarian macrophages found in *Asc*^−/−^ mice could explain the decreased local inflammatory response, which subsequently would contribute to the elevated oocyte survival. A limitation of our flow cytometry study is that the estrous cycle of mice were not synchronized, which can affect the final results since macrophage number and distribution can change during the estrous cycle (Wu et al., 2004). Furthermore, in this study, cells were firstly gated for singlets and traditional lymphoid/myeloid cell populations. Following this gating strategy, we have probably excluded macrophage multinucleated giant cells, which have been associated with chronic inflammation and ovarian aging (Briley et al., 2016), from the flow cytometry analysis, since these cells are much larger.

While macrophages are known to be important for ovarian function, it is not known what role NK cells play. Recently, Gounder et al. (2018) reported an increase in the circulating human NK cell population and a decrease in the proliferation potential upon aging (Gounder et al., 2018). Activated NK cells secrete a large variety of cytokines, including TNF-α, which, as described earlier, is critical for inflammaging and can promote oocyte-mediated apoptosis (Nersesian et al., 2019). In this study, the proportion of NK cells in the ovary of *Asc*^−/−^ mice tended to be lower than WT mice, potentially highlighting that NK cells may contribute to the ovarian inflammatory environment, though further studies would be required to investigate this possibility in more detail.

Overall, the data presented in this study indicate that ablation of the inflammasome prevents age-related depletion of the ovarian reserve by reducing the local inflammatory environment, possible mediated by a reduction in the percentage of ovarian macrophages in aged inflammasome-deficient mice. The results observed in this study significantly advance our understanding of the mechanisms responsible for the regulation of oocyte number as female age and provide potential therapeutic targets for the development of new strategies to improve reproductive lifespan for advanced reproductive-aged women.

## Data Availability Statement

The raw data supporting the conclusions of this article will be made available by the authors, without undue reservation.

## Ethics Statement

The animal study was reviewed and approved by Monash Animal Ethics Committee.

## Author Contributions

KH, AM, and CL designed the experiments, analyzed the data, and edited the manuscript. CL and SL performed the experiments. CL and KH wrote the manuscript. All authors contributed to the article and approved the submitted version.

## Conflict of Interest

The authors declare that the research was conducted in the absence of any commercial or financial relationships that could be construed as a potential conflict of interest.
