# Supplementary material for: The Inflammasome Contributes to Depletion of the Ovarian Reserve During Aging in Mice
Source: Front Cell Dev Biol. 2021 Feb 11;8:628473. doi: 10.3389/fcell.2020.628473 (PMC7905095; doi:10.3389/fcell.2020.628473)
Supplement: Supplementary file 1 [file Presentation_1.PPTX]

## Slide 1
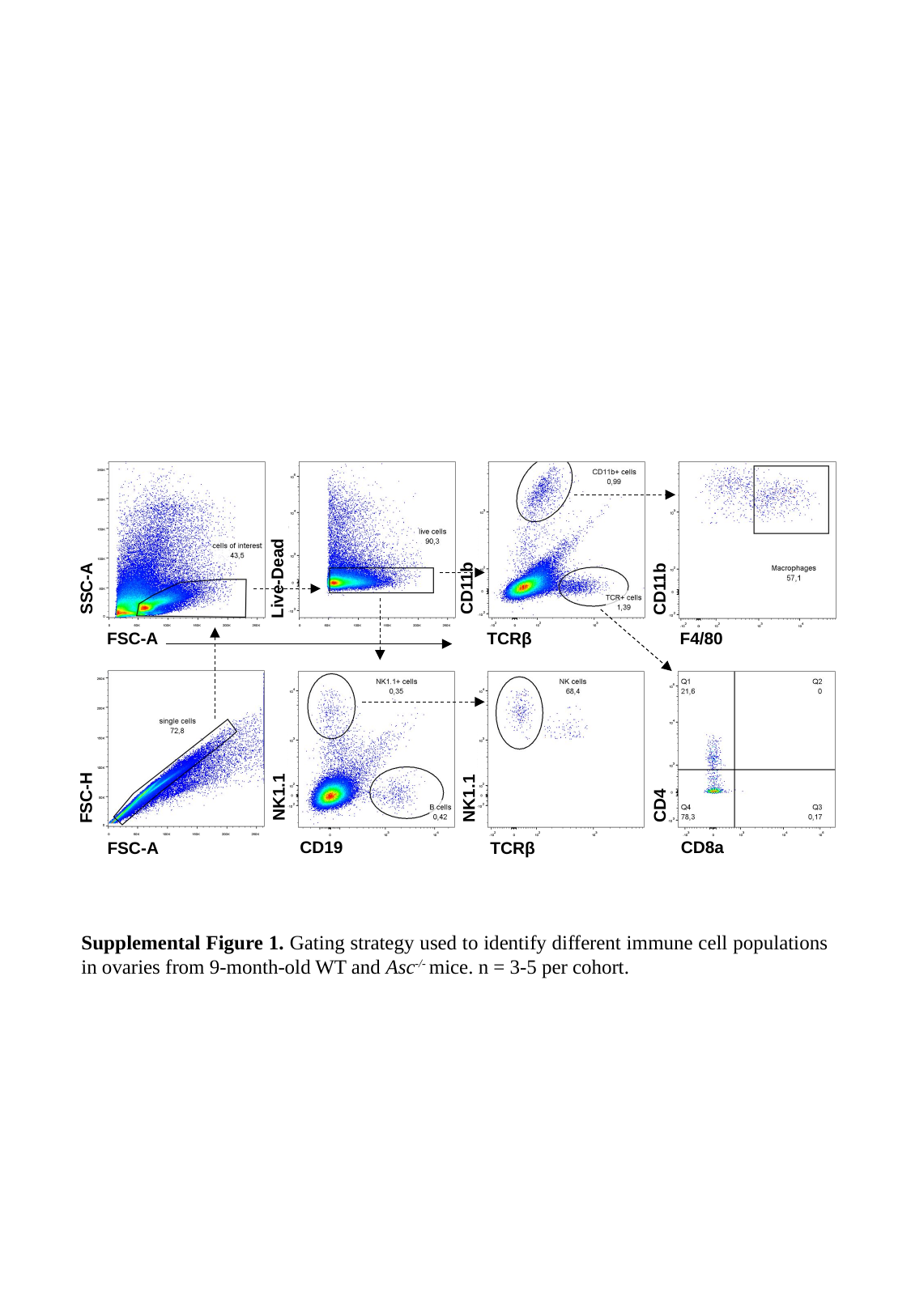

Live-Dead
CD11b
SSC-A
CD11b
TCRβ
FSC-A
F4/80
NK1.1
FSC-H
NK1.1
CD4
CD19
CD8a
FSC-A
TCRβ
Supplemental Figure 1. Gating strategy used to identify different immune cell populations in ovaries from 9-month-old WT and Asc-/- mice. n = 3-5 per cohort.

## Slide 2
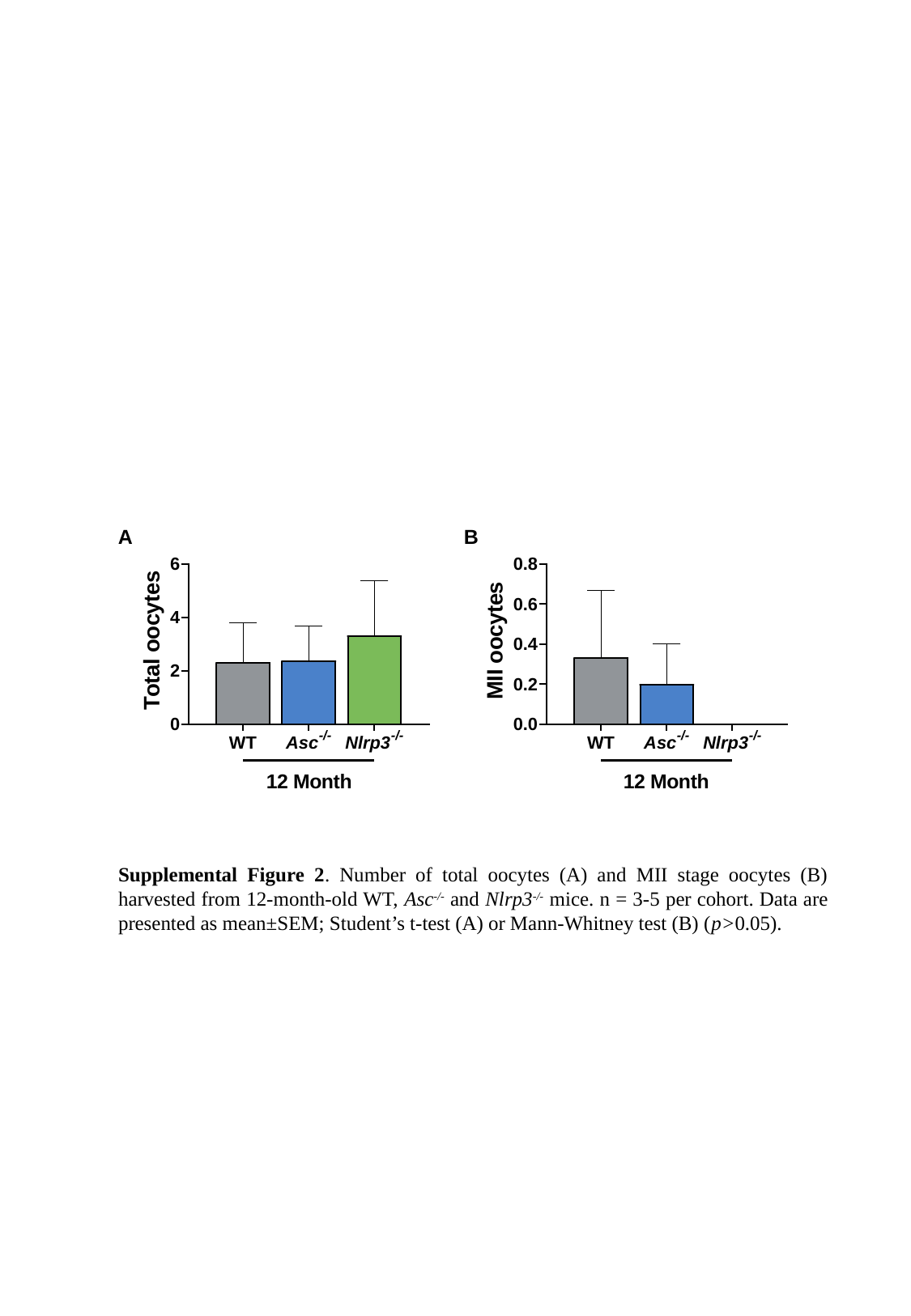

A
B
Supplemental Figure 2. Number of total oocytes (A) and MII stage oocytes (B) harvested from 12-month-old WT, Asc-/- and Nlrp3-/- mice. n = 3-5 per cohort. Data are presented as mean±SEM; Student’s t-test (A) or Mann-Whitney test (B) (p>0.05).

## Slide 3
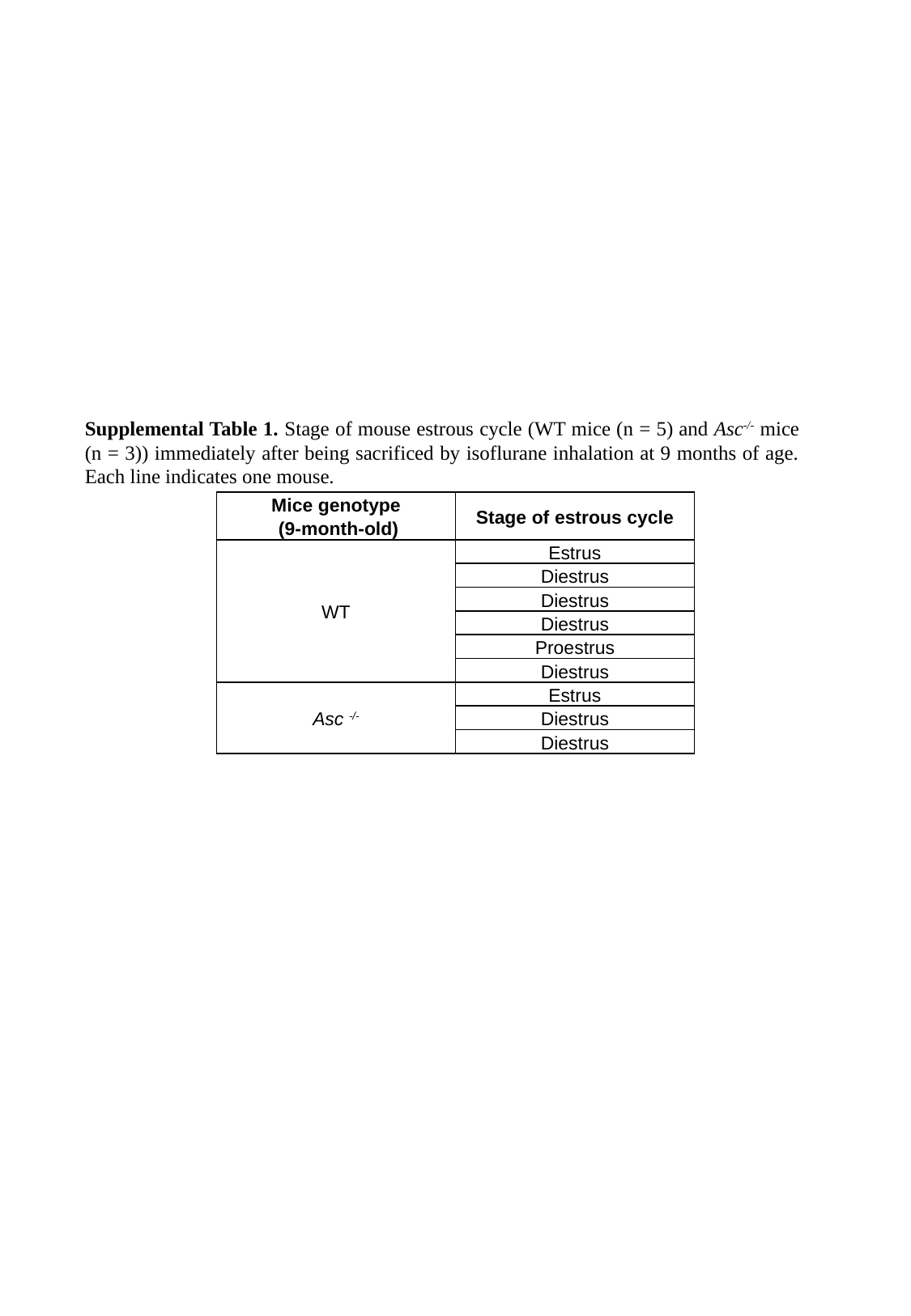

Supplemental Table 1. Stage of mouse estrous cycle (WT mice (n = 5) and Asc-/- mice (n = 3)) immediately after being sacrificed by isoflurane inhalation at 9 months of age. Each line indicates one mouse.
| Mice genotype (9-month-old) | Stage of estrous cycle |
| --- | --- |
| WT | Estrus |
| | Diestrus |
| | Diestrus |
| | Diestrus |
| | Proestrus |
| | Diestrus |
| Asc -/- | Estrus |
| | Diestrus |
| | Diestrus |

## Slide 4
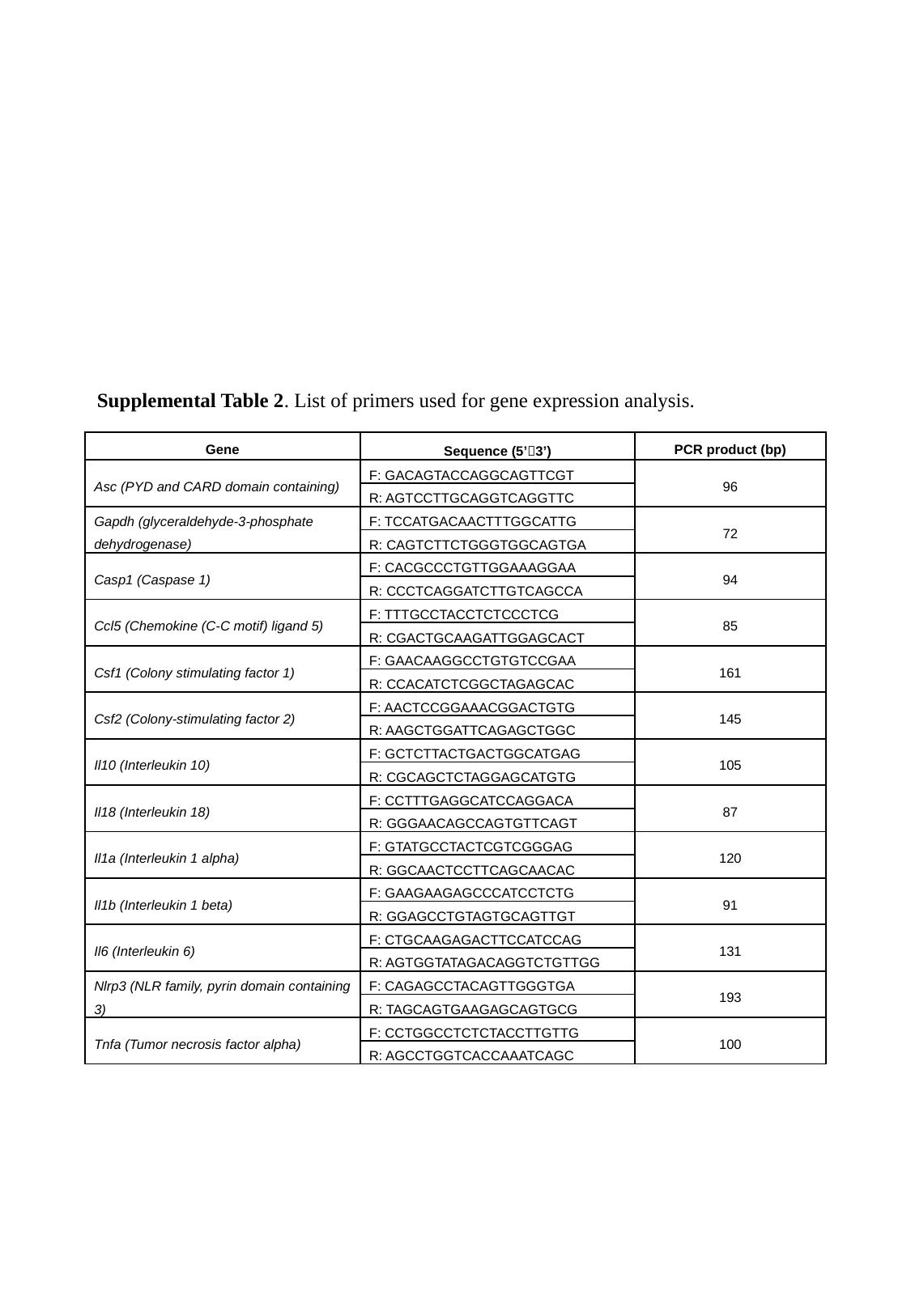

Supplemental Table 2. List of primers used for gene expression analysis.
| Gene | Sequence (5’3’) | PCR product (bp) |
| --- | --- | --- |
| Asc (PYD and CARD domain containing) | F: GACAGTACCAGGCAGTTCGT | 96 |
| | R: AGTCCTTGCAGGTCAGGTTC | |
| Gapdh (glyceraldehyde-3-phosphate dehydrogenase) | F: TCCATGACAACTTTGGCATTG | 72 |
| | R: CAGTCTTCTGGGTGGCAGTGA | |
| Casp1 (Caspase 1) | F: CACGCCCTGTTGGAAAGGAA | 94 |
| | R: CCCTCAGGATCTTGTCAGCCA | |
| Ccl5 (Chemokine (C-C motif) ligand 5) | F: TTTGCCTACCTCTCCCTCG | 85 |
| | R: CGACTGCAAGATTGGAGCACT | |
| Csf1 (Colony stimulating factor 1) | F: GAACAAGGCCTGTGTCCGAA | 161 |
| | R: CCACATCTCGGCTAGAGCAC | |
| Csf2 (Colony-stimulating factor 2) | F: AACTCCGGAAACGGACTGTG | 145 |
| | R: AAGCTGGATTCAGAGCTGGC | |
| Il10 (Interleukin 10) | F: GCTCTTACTGACTGGCATGAG | 105 |
| | R: CGCAGCTCTAGGAGCATGTG | |
| Il18 (Interleukin 18) | F: CCTTTGAGGCATCCAGGACA | 87 |
| | R: GGGAACAGCCAGTGTTCAGT | |
| Il1a (Interleukin 1 alpha) | F: GTATGCCTACTCGTCGGGAG | 120 |
| | R: GGCAACTCCTTCAGCAACAC | |
| Il1b (Interleukin 1 beta) | F: GAAGAAGAGCCCATCCTCTG | 91 |
| | R: GGAGCCTGTAGTGCAGTTGT | |
| Il6 (Interleukin 6) | F: CTGCAAGAGACTTCCATCCAG | 131 |
| | R: AGTGGTATAGACAGGTCTGTTGG | |
| Nlrp3 (NLR family, pyrin domain containing 3) | F: CAGAGCCTACAGTTGGGTGA | 193 |
| | R: TAGCAGTGAAGAGCAGTGCG | |
| Tnfa (Tumor necrosis factor alpha) | F: CCTGGCCTCTCTACCTTGTTG | 100 |
| | R: AGCCTGGTCACCAAATCAGC | |

## Slide 5
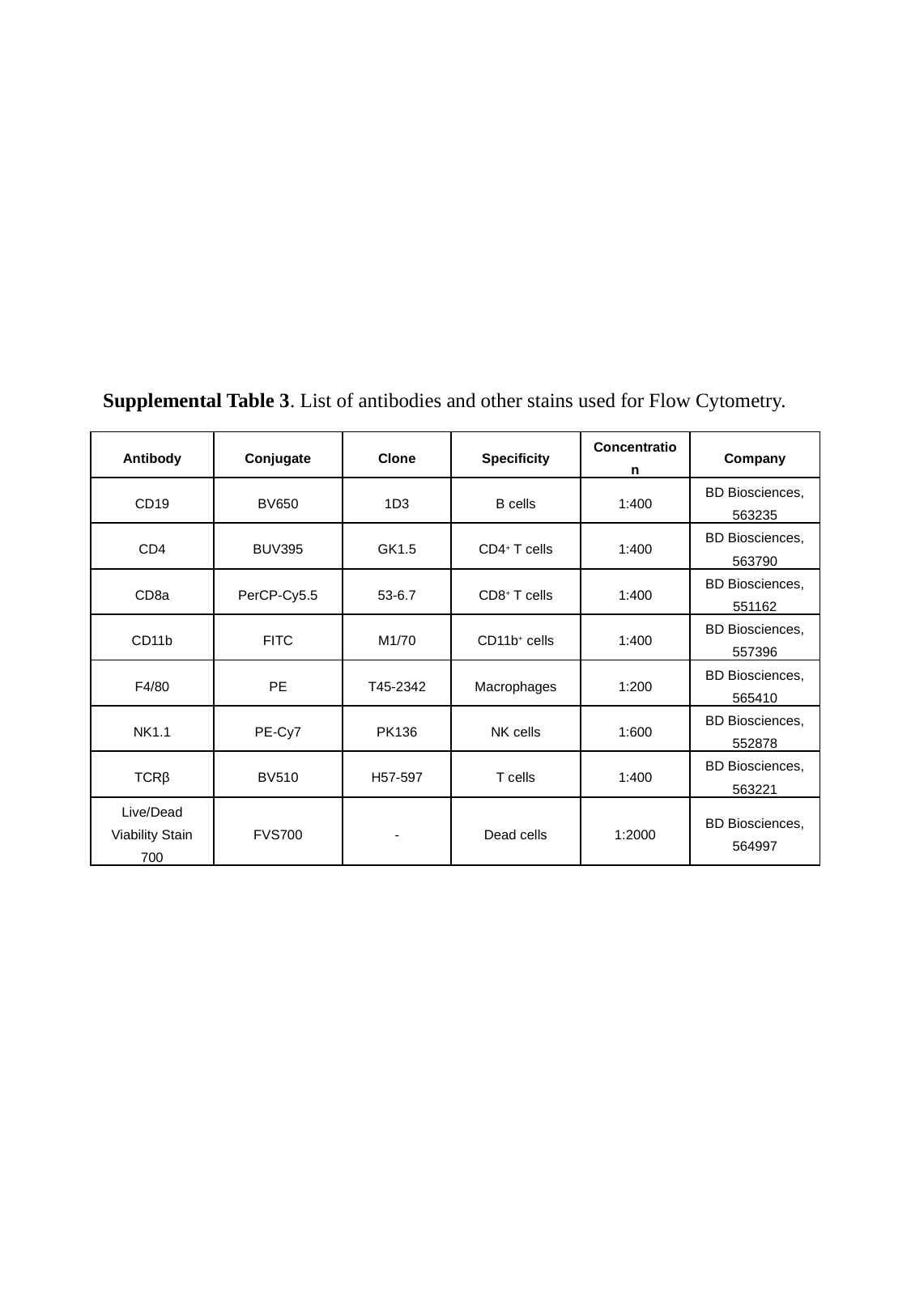

Supplemental Table 3. List of antibodies and other stains used for Flow Cytometry.
| Antibody | Conjugate | Clone | Specificity | Concentration | Company |
| --- | --- | --- | --- | --- | --- |
| CD19 | BV650 | 1D3 | B cells | 1:400 | BD Biosciences, 563235 |
| CD4 | BUV395 | GK1.5 | CD4+ T cells | 1:400 | BD Biosciences, 563790 |
| CD8a | PerCP-Cy5.5 | 53-6.7 | CD8+ T cells | 1:400 | BD Biosciences, 551162 |
| CD11b | FITC | M1/70 | CD11b+ cells | 1:400 | BD Biosciences, 557396 |
| F4/80 | PE | T45-2342 | Macrophages | 1:200 | BD Biosciences, 565410 |
| NK1.1 | PE-Cy7 | PK136 | NK cells | 1:600 | BD Biosciences, 552878 |
| TCRβ | BV510 | H57-597 | T cells | 1:400 | BD Biosciences, 563221 |
| Live/Dead Viability Stain 700 | FVS700 | - | Dead cells | 1:2000 | BD Biosciences, 564997 |
